# Modulation of Cellular Function by the Urokinase Receptor Signalling: A Mechanistic View

**DOI:** 10.3389/fcell.2022.818616

**Published:** 2022-04-08

**Authors:** Daniela Alfano, Paola Franco, Maria Patrizia Stoppelli

**Affiliations:** Institute of Genetics and Biophysics “Adriano Buzzati-Traverso”, National Research Council, Naples, Italy

**Keywords:** urokinase receptor, cell migration, epithelial mesenchyme transition, stemness, cell differentiation, virus infection

## Abstract

Urokinase-type plasminogen activator receptor (uPAR or CD87) is a glycosyl-phosphatidyl-inositol anchored (GPI) membrane protein. The uPAR primary ligand is the serine protease urokinase (uPA), converting plasminogen into plasmin, a broad spectrum protease, active on most extracellular matrix components. Besides uPA, the uPAR binds specifically also to the matrix protein vitronectin and, therefore, is regarded also as an adhesion receptor. Complex formation of the uPAR with diverse transmembrane proteins, including integrins, formyl peptide receptors, G protein-coupled receptors and epidermal growth factor receptor results in intracellular signalling. Thus, the uPAR is a multifunctional receptor coordinating surface-associated pericellular proteolysis and signal transduction, thereby affecting physiological and pathological mechanisms. The uPAR-initiated signalling leads to remarkable cellular effects, that include increased cell migration, adhesion, survival, proliferation and invasion. Although this is beyond the scope of this review, the uPA/uPAR system is of great interest to cancer research, as it is associated to aggressive cancers and poor patient survival. Increasing evidence links the uPA/uPAR axis to epithelial to mesenchymal transition, a highly dynamic process, by which epithelial cells can convert into a mesenchymal phenotype. Furthermore, many reports indicate that the uPAR is involved in the maintenance of the stem-like phenotype and in the differentiation process of different cell types. Moreover, the levels of anchor-less, soluble form of uPAR, respond to a variety of inflammatory stimuli, including tumorigenesis and viral infections. Finally, the role of uPAR in virus infection has received increasing attention, in view of the Covid-19 pandemics and new information is becoming available. In this review, we provide a mechanistic perspective, via the detailed examination of consolidated and recent studies on the cellular responses to the multiple uPAR activities.

## Introduction

### Urokinase Receptor Structure and Ligands

The uPAR is a glycosylphosphatidyl-inositol (GPI)-anchored membrane protein belonging to the Ly-6 (Lymphocyte antigen-6) protein family. This receptor was first identified for its high affinity and specificity binding to the amino-terminal fragment of urokinase (ATF, residues 1–135) on blood monocytes and on the U937 monocyte-like cell line (Stoppelli et al., 1985; Vassalli et al., 1985). The uPAR was initially regarded as a binding site to drive plasminogen-dependent pericellular matrix degradation, but subsequently found to be a true signal transducer with the ability to modulate cellular functions, including proliferation, survival, migration and invasion (Blasi and Carmeliet 2002).

### Glycosyl-Phosphatidyl-Inositol Anchored and Soluble Urokinase Receptor Forms

The uPAR is including as a 335 residues single polipeptide chain, including a 22-residue N-terminal signal peptide, and reduced to a 283 residues mature product, upon removal of the N-terminal and C-terminal signal peptides and addition of a preformed GPI anchor (Roldan et al., 1990). The polypeptide backbone of uPAR has an apparent molecular mass of 35 kDa, but the mature uPAR molecule has a higher molecular mass of ∼55 kDa, due to its extensive and heterogeneous glycosylation (Behrendt et al., 1990).

**FIGURE 1:**
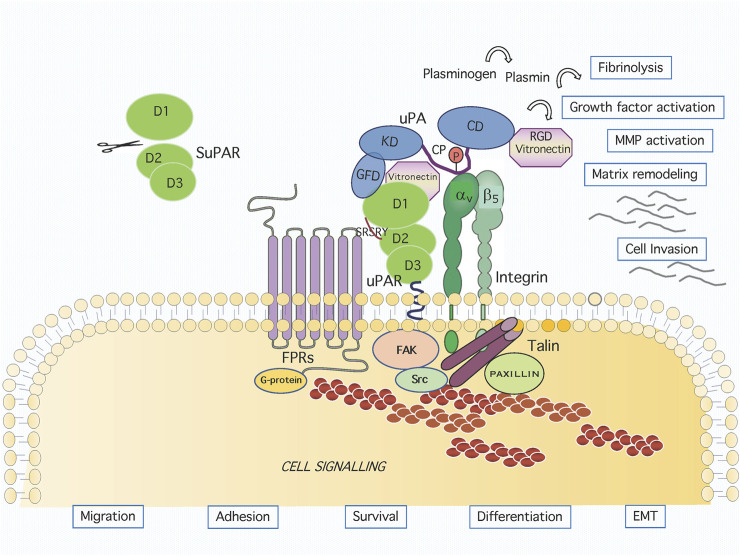
Graphic representation of the uPAR system on cell surface. Active urokinase (uPA) catalyses the conversion of the inactive zymogen plasminogen to the active proteinase plasmin, which can degrade most extracellular matrix proteins. The uPA domains are represented as the growth factor-like domain (GFD, residues 1–49), the kringle domain (KD, residues 50–131), the interdomain linker or “connecting peptide” (CP, residues 132–158), and the serine protease domain (CD, residues 159–411). The uPA interacts with the GPI-anchored urokinase receptor (uPAR) through GFD and with integrin through CP, bridging the two receptors together. The uPAR consists of three homologous domains denoted D1 (residues 1–92), D2 (residues 93–191) and D3 (residues 192–283). The uPAR/integrin interaction results in the regulation of focal contacts turnover. Association and signalling through formyl peptide receptors (FPRs) involves the chemotactic SRSRY uPAR sequence. Membrane proximally interactions result in intracellular signaling and the regulation of migration, adhesion, survival, differentiation and EMT.

The GPI moiety consists of phosphatidylinositol linked to an unusual non-N-acetyl glucosamine, which, in turn, is linked to three mannose residues followed by an ethanolamine covalently linked to the protein. Following biosynthesis, the preformed anchor is attached to the protein by a GPI transamidase, cleaving the peptide bond at the GPI-anchor attachment site and creating an amide linkage between the ethanolamine of the GPI and the newly generated carboxyl group in the precursor protein (Mayor and Riezman 2004). Unlike the transmembrane proteins spanning the entire bilayer, the uPAR is anchored to the outer leaflet of cell membrane and lacks a signal transducing cytoplasmic tail. Nevertheless, it is able to profoundly affect cellular behavior (Figure 1).

The protein moiety consists of three Ly6/uPAR/alpha-neurotoxin-like (LU) homologous domains denoted D1 (residues 1–92), D2 (residues 93–191) and D3 (residues 192–283). Cleavage of the GPI anchor by phospholipases, alternative splicing of uPAR mRNA or proteolytic cleavage of the surface receptor may generate truncated forms of uPAR, either soluble (SuPAR) or membrane-associated. The SuPAR may, in turn, undergo proteolytic cleavage of the linker between D1 and D2 domains, thus generating free D1 and D2D3 domains (Hoyer-Hansen et al., 1992; Sidenius and Blasi 2000). Recently, Moolenaar’s group identified the transmembrane glycerophosphodiesterase GDE3 as the first mammalian GPI-specific phospholipase C (GPI-PLC) that cleaves the anchor and releases the GPI-receptor in a soluble form (van Veen et al., 2017).

The D1-D2 linker region includes the minimal ^88^Ser-Arg-Ser-Arg-Tyr^92^ sequence, relevant to the motogen and pro-angiogenic activities of both uPAR and SuPAR (Fazioli et al., 1997; Bifulco et al., 2014). Synthetic peptides corresponding to the linker region display potent pro-migratory and signalling abilities (Fazioli et al., 1997; Bifulco et al., 2012). In this sequence, Ser^90^ is crucial to the uPAR chemotactic activity, as the S90E mutation inactivates the receptor, whereas the S90P substitution enhances uPAR activity (Bifulco et al., 2012). Based on the conformational analysis of the uPAR_88–92_ sequence, new peptides (pERERY, RERY, and RERF) were developed that inhibit signalling triggered by uPAR_88–92_ and by Vascular Endothelial Growth Factor (VEGF) (Bifulco et al., 2013).

### Urokinase and Vitronectin as Urokinase Receptor Ligands

Urokinase (uPA) is secreted as a single-chain, 411 residues zymogen form (pro-uPA) that becomes activated by a single proteolytic cleavage at Lys ^158^-Ile^159^, thus generating a two-chain molecule linked by a disulfide bridge between Cys^148^ and Cys^279^. The active enzyme catalyses the conversion of the inactive zymogen plasminogen to the active proteinase plasmin, which can degrade most extracellular matrix proteins. Pro-uPA consists of a growth factor-like domain (GFD, residues 1–49), a kringle domain (KD, residues 50–131), an interdomain linker or “connecting peptide” (CP, residues 132–158), and a serine protease domain (residues 159–411) (Figure 1). Two-chains uPA can be further cleaved, releasing an amino-terminal fragment (ATF, residues 1–135) and a small region linked to a large C-terminal proteolytic domain (Alfano et al., 2005). The ATF or the GFD bind to the uPAR D1 domain and binding ability is retained by a peptide encompassing the 18–32 residues of human uPA (Appella et al., 1987; Reuning et al., 2003; Carriero and Stoppelli 2011). The crystal structure of uPAR in complex with a synthetic antagonist peptide or with ATF has revealed a large hydrophobic cavity assembled by the three uPAR domains where the ß-hairpin region of GFD binds (Huai et al., 2006). Structural studies conducted using the SuPAR have shown that the D2 domain is contacted by the amino-terminal region of uPA through one stretch of residues (Ser^21^, Asn^22^, Lys^23^, and Tyr^24^). A second region, highly contributing to the high-affinity uPAR-uPA interaction, is formed mainly by D1 residues interacting with Phe^25^, Ile^28^, and Trp^30^ uPA residues. The third region consists of hydrogen bonds and van der Waals contacts of D1 domain with ATF and Kringle residues (Huai et al., 2006). Functional and structural evidence shows that, upon uPA binding, a conformational change favors the subsequent binding of the matrix protein Vitronectin (Vn) to a region located at the interface between D1 and D2 domains. The affinity binding for Vn is enhanced by uPAR engagement with the uPA GFD, that leads to a structural transition involving the flexible uPAR N-terminal domain (Mertens et al., 2012; Leth et al., 2019). A complete alanine scan of the uPAR showed that all the mutants impaired in the control of cell morphology and migration exhibit impaired Vn binding, thus highlighting the functional relevance of this interaction (Madsen et al., 2007). The uPAR-Vn interaction is entirely mediated by the D1-D2 interface of uPAR, with the residues Trp^32^, Arg^58^, Ile^63^ (in DI), Arg^91^ and Tyr^92^ (in the D1-D2 linker), being crucial both for binding and for the Vn-dependent biological effects (Gårdsvoll and Ploug 2007). Binding of Vn to uPAR occurs via somatomedin B domain (SBD, residues 1–44) (Deng et al., 1996; Deng et al., 2001). The crystal structures of uPAR in complex with ATF and SBD confirm that uPAR may concomitantly engage these two ligands and reveal that the uPA epitope occupies the central cavity of the receptor, whereas Vn binds on the receptor outer side (Huai et al., 2008). Mechanistically, binding of uPA drives uPAR into its closed conformation, corresponding to a high affinity state for Vn, thus leading to an allosteric regulatory mechanism (Zhao et al., 2015). Secreted uPA may be phosphorylated on Ser^138^ located in the CP region and Ser^303^ in the catalytic domain. This modification does not alter the protease binding affinity for uPAR but impairs uPA chemotactic ability (Franco et al., 1997). Phosphorylation occurs in A431 human carcinoma cells prior to secretion and is down regulated by protein kinase C inhibitors (Franco et al., 1998). Further evidence showing the functional relevance of uPA CP region is based on the effects of Å6, a peptide corresponding to uPA residues 135–143 which inhibits tumour progression and angiogenesis (Guo et al., 2000). Further studies of the CP uncovered the 144–158 region and derived peptides, endowed with a clearcut motogen activity (Franco et al., 2013).

Early evidence showed that receptor-bound uPA retains the ability to activate plasminogen (Ellis et al., 1991). In turn, plasminogen may be bound to cell surface through receptors denoted Plg-Rkt and promote plasminogen activation in association with uPA/uPAR complexes (Miles and Parmer 2013; Miles et al., 2020). The concomitant activity of surface-bound uPA and plasmin may result also in the cleavage of the RGD motif in Vn, negatively regulating uPAR-mediated cell adhesion to Vn (De Lorenzi et al., 2016).

## Membrane Interactors and Proximal Signalling

GPI-receptors are embedded in the leaflet of membranes through their glycolipid moieties and are not accessible from the cytosolic side of the membrane. However, they physically associate with transmembrane receptors and cytoplasmic signalling mediators in functional units. A large body of evidence indicates that uPAR physically associates with transmembrane receptors, forming complexes active in signal transduction (Table 1) (Blasi and Carmeliet 2002; Alfano et al., 2005; D’Alessio and Blasi 2009; Smith and Marshall 2010).

**TABLE 1:** Membrane interactors of uPAR and signalling effects.

uPAR signalling partners	Specimen	Type of analysis	Functional outcome	References
Integrins αvβ1/β3/β5 α5β1/α3β1	Breast, fibrosarcoma, oral squamous carcinomas and gliomas, podocytes/kidney cells cephalic explants cultures	In culture, ex vivo, in vivo	p130Cas, FAK and Src phosphorylation; cell migration; cell adhesion to ECM; cell-cell adhesion tumor growth and invasion kidney disfunction neurite outgrowth and neuritogenesis	Xue et al., 1997; Carriero et al., 1999; Nguyen and Hildreth, 2000; Aguirre Ghiso et al., 1999; Aguirre Ghiso, 2002; Zhang, 2003; Wei et al., 2005; Gargiulo et al., 2005; Wei et al., 2008; Malla et al., 2013; Lino et al., 2014; Eden et al., 2018
FPR1	Melanoma, ovarian, prostate and lung carcinoma, sarcoma	In culture, in vivo	Partitioning of uPAR to lipid rafts; increased cell migration and invasion; vessel sprouting; increased intra-tumoral microvessel density; reduction of tumor size; increased circulating tumor cells and pulmonary metastases	Bifulco et al., 2008; Carriero et al., 2009; Montuori et al., 2011; Rea et al., 2013; Bifulco et al., 2013; Bifulco et al., 2014; Gorrasi et al., 2014; Ragone et al., 2017; Carriero et al., 2017; Minopoli et al., 2019
LRP	Breast cancer, Prostate cancer	In vitro, in culture	Clathrin-dependent pathway activation; endocytosis of the uPA-PAI-2 and uPA-PAI-1 complexes; surface plasmin generation and matrix invasion; tumor growth inhibition	Czekay et al., 2001; Gonias, 2001; Croucher et al., 2006; Miller-Kleinhenz et al., 2018
CXCR4	Kidney ephitelial cells	In culture	Cell migration; cell adhesion	Montuori et al. (2011)
EGFR	Glioblastoma, squamous carcinoma	In culture, in vivo	PKC/integrin signaling; tumor cell survival and growth; tumor dormancy	Liu et al., 2002; Mazzieri et al., 2006; Hu et al., 2011; Alapati et al., 2012; Hu et al., 2015; Wykosky et al., 2015; Eden et al., 2018
PDGFR	Vascular smooth muscle cells; mesenchymal stem cells, macrophages	In culture	Cell migration; cell proliferation cholesterol biosynthesis	Kiyan et al., 2005; Fuhrman et al., 2009; Chabot et al., 2015
VEGFR2	Endothelial cells	In culture, in vivo	Angiogenesis; neovascularization	Herkenne et al. (2015)
IGFR1	Breast cancer	Ex vivo	Cell invasion and metastasis	Huber et al. (2016)
sLR11	Hematopoietic stem and progenitor cells	In vitro, ex vivo	Adhesion of HSPCs to bone marrow niche	Nishii et al. (2013)
Caveolin	Lung fibroblasts, endothelial progenitors vascular smooth muscle cells	In culture, ex vivo	Angiogenesis; vascular remodelling; idiopathic pulmonary fibrosis	Kiyan et al., 2009; Margheri, 2011; Grove et al., 2014; Margheri, 2015

### Integrins

The integrins are heterodimeric adhesion receptors for ECM proteins such as collagen, fibronectin, laminin and Vn, with cytoplasmic domains acting as scaffolds for the assembly of multiprotein signalling complexes linking them to the cytoskeleton (Desgrosellier and Cheresh 2010). Many integrin receptors were shown to associate physically and functionally to the uPAR in cells of different origin, including αLß2, αMß2, αvß5, α5ß1, αvß3, α3ß1 (Table 1) (Carriero and Stoppelli 2011). Many studies have addressed the mechanism as well as the involved regions of the uPAR/integrins interaction. An interesting possibility derives from our studies, conducted on the uPA CP domain, binding to avß5 integrin and promoting cytoskeletal rearrangements and directional cell migration, in the presence of uPAR. As GFD-uPAR and CP-integrin binding are not mutually exclusive, we have proposed a model for uPAR/integrin complex formation in which the concomitant ligand binding bridges the two receptors together (Figure 1). Interestingly, this binding is not dependent on the GFD-uPAR interaction, and is retained by the 135–158 peptide (CPp). This peptide binds to αvβ5 integrin with high affinity, induces chemotaxis at picomolar concentrations, and stimulates the association of uPAR and αvβ5 integrin (Franco et al., 2006).

In HT1080 fibrosarcoma cells, αvß5 co-purifies with uPAR and the uPAR/αvß5 complexes drive cell migration and cytoskeletal rearrangements (Carriero et al., 1999). In fibrosarcoma cells, uPAR co-clusters also with ß1 and ß3 integrin subunits, as shown by resonance energy transfer studies (Xue et al., 1997). To assess direct uPAR/integrin interaction, synthetic peptides and receptor variants have been employed in several successful studies. The involvement of the α3 *ß*-propeller (residues 242–246) in the α3β1/uPAR interaction is shown by two Ala substitutions (H245A or R244A) in α3, disrupting receptor association. Also, integrin-derived α325 peptide, corresponding to residues 241–257, binds to SuPAR, supporting a direct uPAR-α3 chain interaction (Zhang et al., 2003). Another peptide, derived from the *ß*-propeller region of αM integrin and named M25 binds to uPAR and disrupts its association with ß1 and ß2 subunits in monocytic cells (Simon et al., 2000). However, direct uPAR/integrin binding is not restricted to the α chains, as the S227A point mutation in the ß1 chain and a peptide corresponding to a ß1 sequence near the known α-chain uPAR-binding region abrogate uPAR/a5ß1 complex formation (Wei et al., 2005). Regarding the uPAR region involved in the association with integrins, Degryse et al. identified the GAAG sequence (residues 133–136 in D2 domain) critical to the association with a3ß1. First of all, the uPAR D262A variant fails to associate with a3ß1 and D2-derived specific peptides and induce a3ß1signalling (Degryse et al., 2005). Further work by Chaurasia et al. showed that a peptide encompassing residues 240–248 of uPAR DIII domain binds to purified a5ß1. Also, the S245A substitution in this peptide or in the full GPI-anchored uPAR prevents its association to a5ß1 (Chaurasia et al., 2006).

In general, the association of uPAR with integrins elicits various cellular effects, including changes in cell adhesion, migration and signalling (Alfano et al., 2005; Smith and Marshall 2010). The α5ß1 integrin/uPAR interaction results in RGD-independent, but uPAR-dependent adhesion to fibronectin, suggesting that the complexed α5ß1 integrin acquires distinct functional properties (Wei et al., 2005). In uPA-treated MCF-7 breast carcinoma cells, uPAR/αvß5 association directs cytoskeletal rearrangements and cell migration through PKC activity, whereas binding of Vn to αvß5 results in distinct, PKC-independent effects (Carriero et al., 1999). In human HEp3 squamous cell carcinoma growth *in vivo*, the extent of uPAR/αvß5 physical association is important to maintain high levels of ERK1/2 activity, whereas the inhibition of uPAR expression leads to a reduced complex formation and tumour dormancy (Aguirre Ghiso et al., 1999). Further details are provided by Ferrari et al., showing that uPAR-mediated cell adhesion to Vn triggers a novel type of ligand-independent integrin signalling, occurring also with integrins deficient in ligand binding (Ferraris et al., 2014).

### Formyl Peptide Receptors

Among the uPAR cis-acting receptors, the formyl peptide receptors or FPRs have been the subject of intense investigation (Table 1). FPRs are seven transmembrane G protein-coupled receptors displaying high affinity for N-formyl peptides, such as formyl-Met-Leu-Phe (fMLP), naturally released by bacteria. Of the three FPRs identified in humans, FPR and FPRL-1 are highly expressed in blood monocytes and neutrophils, whereas FPRL-2 is expressed in monocytes, eosinophil and dendritic cells (Gavins 2010). Resnati and coworkers showed that FPRL1 is required for the uPA/uPAR-dependent chemotaxis and that direct binding of isolated uPAR D2D_388-274_ to FPRL1 is competed by two specific FPRL1 agonists, the synthetic MMK-1 peptide and a stable lipoxin analog (Resnati et al., 2002). Further information was generated by the use of a peptide corresponding to the uPAR_84-95_ region, shown to be a potent chemoattractant for basophils following specific binding to FPRL1 and FPRL2 (de Paulis et al., 2004). Following uPAR engagement with uPA, a conformational transition results in the exposure of the uPAR_88-92_ sequence that can participate in the interaction with co-receptors (Figure 1). The intricate functional relationship of uPAR and FPRs has been described by several investigators. First, exposure of monocytes to increasing amounts of uPAR_88-274_ prevents migration in response to MCP-1 (monocyte chemoattracting protein-1), RANTES and fMLP (Furlan et al., 2004). Among the signalling events triggered by the uPAR_88-92_ sequence (SRSRY) are increased directional migration, remarkable cytoskeletal rearrangements and ERK1/2 phosphorylation, all inhibited by FPR desensitization with high concentrations of fMLP (Gargiulo et al., 2005). The functional crosstalk between uPAR and CXCR4 was also confirmed by showing their co-regulation through a common microRNA in acute myeloid leukemia (Alfano et al., 2015). The involvement of αv integrins together with fMLP receptors and uPAR is shown in CXCR4-expressing cells migrating toward stromal-derived factor-1 (Montuori et al., 2011). As shown by Gorrasi et al., also β1 integrins may be complexed with uPAR and FPR1 at the HEK-293 embryonic kidney cell surface, thus driving pro-migratory signalling (Gorrasi et al., 2014). The functional interaction of uPAR with fMLP receptors and integrins is reported to be critical for the capability of uPAR to regulate uPA expression (Montuori et al., 2013).

Extensive work was devoted to define the functional properties of the uPAR_88-92_ sequence, that are maintained even in the form of SRSRY peptide, endowed with strong chemotactic properties for a variety of different cell types. In contrast, the Ser to Glu-substituted, ERERY peptide is a strong inhibitor of SRSRY-directed cell migration. Interestingly, the ERERY peptide competes with fMLF for binding to FPR, paving the way to the design of novel anti-metastatic compounds (Bifulco et al., 2008). Among the SRSRY-derived inhibitors of cell migration and invasion by a drug design approach is Ac-Arg-Glu-Arg-Phe-NH_2_ (RERF), preventing not only SRSRY-directed cell migration, but causing also a 3- to 5-fold reduction of lung metastasis number and size in nude mice following caudal injection of HT1080 cells (Carriero et al., 2009). Novel molecules targeting S88 and R91, located in the chemotactic sequence, also inhibit the interaction between uPAR and FPR1, and block migration and invasion toward FBS, uPA and fMLF (Rea et al., 2013).

More recently, Carriero’s group showed that the association of uPAR with the overexpressed FPR1 leads to the melanoma and ovarian cancer cell invasion, that is inhibited by the potent RI-3 peptide, disrupting the uPAR_84–95_/FPR1 interaction (Ragone et al., 2017; Minopoli et al., 2019). Following the subcutaneous injection of sarcoma cells in nude mice, administration of the RI-3 peptide results in the reduction of tumor size, intra-tumoral microvessel density, circulating tumor cells and pulmonary metastases (Carriero et al., 2017). The crosstalk of FPRs with the uPA/uPAR system is not limited to neoplastic conditions, but it also modulates the redox state in systemic sclerosis chronic autoimmune disease (Napolitano et al., 2018).

### Growth Factor Receptors

Many studies address the physical and functional association of uPAR with the epidermal growth factor receptor (EGFR) (Table 1). Liu et al. showed that uPAR overexpression leads to uPAR/EGFR co-immunoprecipitation and concomitant activation of EGFR tyrosine autophosphorylation, even in the absence of EGF, uncovering an intricate cross-talk between the two receptors (Liu et al., 2002). Jo et al. showed that uPA promotes Chinese hamster ovary CHO-K1 cell proliferation, exclusively in the EGFR-expressing cells and requires activation of STAT5b and ERK (Jo et al., 2005). The hypothesis that uPAR is required for EGFR activation is supported by D'Alessio et al., showing that, in uPAR deficient mouse keratinocytes, EGFR signalling activity is essentially lost, even though the expression level of EGFR and EGF is unchanged (D’Alessio et al., 2008). More recently, it was shown that the uPAR D2 includes a motif (D2A) with a particular three-dimensional structure, promoting EGFR phosphorylation and EGFR-dependent cell proliferation, thereby confirming the interdependence of uPAR and EGFR (Eden et al., 2018). In glioblastoma multiforme (GBM), the uPAR interacts with a truncated variant of EGFR, supporting tumor cell survival and growth (Hu et al., 2011; Hu et al., 2015). Furthermore, in a model system of GBM acquired resistance, the uPA/uPAR signalling is required for the repression of the Bim proapoptotic factor, thus promoting resistance to EGFR tyrosine kinase inhibitors (Wykosky et al., 2015). A very recent study provided new insights into the control of EGFR expression by melanoma-derived, uPAR-expressing exosomes. Following uPAR genetic deletion by CRISPR/Cas9, the EGFR expression decreases and the pro-tumoral and the pro-angiogenic effects of these vesicles are reduced (Biagioni et al., 2021). Among tyrosine kinase receptors known to interact with the uPAR is the Platelet Derived Growth Factor receptor (PDGFR). Ligand-engagement of uPAR induces its association with PDGFR-ß, receptor dimerization, PDGF-independent phosphorylation of the cytoplasmic domain, and uPA-dependent signalling that regulates vascular smooth muscle cell migration and proliferation (Kiyan et al., 2005). The uPAR participates to the regulation of endothelial cell migration and invasion induced by VEGF165, VEGF-E, Fibroblast Growth Factor-2 (FGF-2), EGF and Hepatocyte Growth Factor (HGF), associated to surface pro-uPA activation and uPAR redistribution (Poettler et al., 2012). Furthermore, the functional link of uPAR with PDGFR and ß1-integrins is uncovered by the findings that receptor association with ß1-integrins is required for PDGFR-induced migration of human mesenchymal stem cells derived from bone marrow and from adipose tissue (Chabot et al., 2015). The interaction of uPAR with VEGF2 is crucial to vascular formation, as uPAR deficiency in mice prevents VEGF-induced angiogenesis (Herkenne et al., 2015).

It is known that uPA may associate to its inhibitor PAI-1 (plasminogen activator inhibitor type-1) and that uPA/PAI-1 complexes may bind to the uPAR and internalized via a mechanism involving the low density lipoprotein receptor-related protein (LRP-1). The uPAR/LRP-1 association occurs through uPAR D3 domain and is essential for uPAR regeneration, surface plasmin production and matrix invasion (Czekay et al., 2001). Further evidence that uPA/PAI-1 complexes induce surface uPAR downregulation and recycling is provided by Gonias et al., showing the LRP-dependence and the involvment of clathrin-coated pathway (Gonias et al., 2011). Similarly, inhibition of uPA by PAI-2 significantly increases the affinity of the complex for LRP, resulting in endocytosis of the uPA-PAI-2 complexes in prostate cancer cells (Croucher et al., 2006). These findings suggest the co-targeting of LRP/uPAR by nanoparticle-drug delivery into breast cancer patient derived xenograft tumors, thus resulting in remarkable tumor growth inhibition (Miller-Kleinhenz et al., 2018).

### Membrane Lipids

GPI-anchored receptors partition preferentially in dynamic membrane domains that are enriched in sphingolipids and cholesterol, and are denoted rafts or detergent-resistant membranes (Simons and Ikonen 1997). Due to the tight packing of sphingolipids, lipid rafts are believed to be highly ordered and less fluid than the surrounding phospholipid bilayer. It has been reported that the lipid microenvironment of unengaged uPAR is enriched in sphingomyelin and glycosphingolipids, whereas, following ligand engagement, there is a selective reduction of neutral glycosphingolipids (Sahores et al., 2008) (Table 1). A regulatory role may be exerted by gangliosides in endothelial progenitor cells, as GM1 ganglioside promotes caveolar-raft partitioning of uPAR, enhancing matrix invasion and capillary morphogenesis (Margheri et al., 2015). Cunningham et al. showed that cell surface uPAR dimerizes, partitioning preferentially to detergent-resistant lipid rafts and that binding of Vn occurs preferentially to raft-associated dimeric uPAR, being completely inhibited by cholesterol depletion (Cunningham et al., 2003). The exchange of monomers and dimers and the dynamic localisation of the receptor are regulated also by its association with Vn or with uPA-PAI-1 complexes (Caiolfa et al., 2007). For GPI-anchored proteins, the lipid anchor with its saturated fatty acyl chains determines raft association, where signalling mediators, including transmembrane receptors and tyrosine kinases can selectively be included or excluded. The relevance of rafts to the formation of uPAR signalling complexes is shown by many reports. In human polymorphonuclear neutrophils, the uPAR directly associates with L-selectin to form a signalling complex in lipid rafts (Sitrin et al., 2001). In migrating T cells, the uPAR colocalizes with CXCR4 in GM3-enriched lipid environment at the leading edge, whereas GM1 is concentrated at the opposite edge in the uropod (Gomez-Mouton et al., 2001). In fibroblasts from patients with idiopathic pulmonary fibrosis, uPAR engagement with uPA leads to the recruitment of α5β1 integrin and Fyn kinase to lipid rafts and to a consequent hypermotile phenotype (Grove et al., 2014). In vascular smooth muscle cells, uPAR-dependent morphological changes involve rafts (Kiyan et al., 2009). Furthermore, the co-localisation of uPAR and MMP-9 in lipid rafts is critical to migration and invasion of breast cancer cells (Raghu et al., 2010).

The multiple ways sphingolipids affect uPAR signalling are testified by the findings that GM3, a major raft component, inhibits uPA-induced EGFR phosphorylation by blocking the integrin/EGFR crosstalk and GT1b suppresses both uPA-induced FAK and EGFR activation by preventing α5ß1 integrin activation (Wang et al., 2005). Although the mechanistic aspects await further investigation, the overexpression of deacetylated GM3 (d-GM3) stimulates uPAR/integrin signalling and p38 MAPK activity, promoting migration and invasion of metastatic melanoma cells (Yan et al., 2013). A recent study has further highlighted the importance and the role of the GPI anchor in uPAR-controlled cell migration by connecting β1 integrins and FPRs; in fact, the uPAR promotes pro-migratory signals through its GPI tail, driving and partioning it into lipid rafts (Gorrasi et al., 2020).

## Regulation of Urokinase Receptor Expression: Genetic and Epigenetic Mechanisms

Early studies highlighted the transcriptional modulation of uPAR expression, in malignant cells stimulated by cytokines and tumor promoters or in highly invasive colon cancer cells (Lund et al., 1995; Wang et al., 1994). The finding that a 51-nt protein binding fragment of uPAR mRNA is involved in mRNA turnover and in cycloheximide-induced stabilization suggests that uPAR may be regulated at mRNA stability level (Shetty et al., 1997). A further level of regulation in monocytic cells is represented by the translation efficiency of uPAR protein that is modulated by adhesion-dependent signalling through the eukaryotic translation initiation factor 4E (Mahoney et al., 2001). Furthermore, protein turnover seems to be accelerated by the inefficient addition of uPAR glycolipid moiety (Avila et al., 2011).

A substantial amount of work has been performed to identify the *cis*- and *trans*-acting factors and regulatory sequences controlling uPAR mRNA expression. At molecular level, the basal transcription of the human uPAR gene is driven by a proximal promoter, contained within 180 bp from the major transcription start sites with relatively GC-rich proximal sequences, lacking TATA and CAAT boxes (Soravia et al., 1995). As shown in HCT 116 colon cancer cells, the K-*Ras* gene induces uPAR transcription through the binding of c-Jun, JunD, c-*Fos* and Fra-1 to the AP-1 motif in the uPAR promoter at –184 (Allgayer et al., 1999a). The Ras-related GTPase RalA upregulates uPAR transcription through a similar mechanism involving the c-Jun binding motif at −184 bp as well as ATF2-like AP1-site at −70 bp (Okan et al., 2001). The uPAR gene is transcriptionally regulated also by the activation of Src tyrosine kinase, via the Sp1 transcriptional activator binding to an upstream sequence (−152/−135) (Allgayer et al., 1999b). Zannetti et al. showed that Sp1-binding activity and uPAR levels are coordinately elevated in breast carcinomas as compared to benign lesions and that uPAR engagement by uPA results in a marked up-regulation of Sp1-binding activity followed by an increase of uPAR protein (Zannetti et al., 2000). The tumor hypoxia-induced HIF expression leads to increased uPAR mRNA through four putative HIF binding sites (Büchler et al., 2009). The uPAR is strongly down modulated by c-Myc activation that promotes apoptosis and reduces cell motility, in the absence of Ras, under conditions in which tumorigenesis is repressed (Alfano et al., 2010).

Early work linking uPAR expression to miRNAs (miRs) showed that miR-146a activity decreased MMP-1, uPA, and uPAR expression level, as well as the migratory and invasive activity of LvBr2 metastatic cells (Hwang et al., 2012). Among other contributions describing a correlation between the levels of specific miRs and uPAR, is the report by Sun et al. showing that the miR10b, directly targeting HOXD10, modulates uPAR and MMP-14 levels, ultimately inducing glioma cell invasion (Sun et al., 2011). Supportive evidence shows that in gliomas, mRNA expression levels of RhoC and uPAR, significantly correlate with the expression of miR-10b (Sasayama et al., 2009). Another miRNA, denoted miR-378a-5p modulates the expression of SUFU, FUS-1, and KLF9, as well as STAMBP and HOXD10 genes, upregulating MMP2 and uPAR, two HOXD10 target genes. Overall, the data show that the *in vitro* tumor-promoting functions of miR-378a-5p, are in part mediated by uPAR (Tupone et al., 2020). Moreover, an indirect regulation of uPAR by miR 324-5p through a direct interaction with the RNA binding ELAVL1 was found in colorectal cancer, resulting in a significant reduction of uPA, uPAR, and MMP-9 levels (Gu et al., 2019). We showed a direct interaction of the uPAR 3′UTR with miR-146a, miR-335 and miR-622, resulting in the down-regulation of uPAR and CXCR4 expression in acute myeloid leukemia cell lines (Alfano et al., 2015). Others report that miR-143 directly targets the uPAR 3′-UTR and that this interaction underlies the therapeutic anti-tumor potential of miR-143 replacement therapy in polymeric nanoparticles by systemic treatment of mice bearing subcutaneous PC-3 tumor xenografts (Wach et al., 2019). Co-expression studies of miRs, and their target proteins, by tissue microarrays have shown that in primary prostate tumors miR-143 is localised in stromal cells and uPAR is mainly found in tumor cells, whereas metastatic tissues exhibit miR-143/uPAR co-staining in the cell cytoplasm (Eckstein et al., 2019). Moreover, it was recently reported that uPAR 3′UTR might act as a molecular sponge, recruiting many miRs, thus regulating several pro-tumoral factors, including cathepsins, MMP2, TfR1, vimentin, ICAM-1, IL-8 and HGF in an acute leukemia cell model (Li Santi et al., 2018).

## Cellular Responses and Intracellular Mediators

The uPAR was originally identified as a binding site for the uPA, holding the active enzyme on cell surface for a localized pericellular plasminogen activation (Stoppelli et al., 1985). Later, it was understood that this receptor not only coordinates membrane proteolytic activity, but is also capable of signal transduction leading to different cell responses. Being associated to the outer lipid leaflet, the uPAR connects to the inner cell via the physical and functional association with other membrane receptors, like the integrins and the FPRs (Eden et al., 2011). Large efforts have been concentrated on the study of the pro-migratory effects of the uPA/uPAR interaction (Blasi and Carmeliet 2002). In myelomonocytic cells, the uPAR modulates intracellular p56/59^hck^ tyrosine kinase activity switching cell motility towards adherence, and providing a mutually exclusive mechanism to regulate these properties during monocyte/macrophage differentiation *in vivo* (Chiaradonna et al., 1999). In breast cancer cells, the uPAR is complexed with αvβ5 Vn receptor, promoting ligand-dependent cell migration (Carriero et al., 1999). Mechanistic studies have shown that increased migration results from the interaction of SRSRY active sequence, in the uPAR or in the form of isolated peptide, with fMLP receptors complexed with αvβ5 (Gargiulo et al., 2005). Among the intracellular effectors regulating cytoskeletal and adhesion dynamics, are Rho family GTPases, crucial modulators of cell migration and invasion (Heasman and Ridley 2008). Many reports concern the involvement of Rho small GTPases in uPAR-directed signaling leading to cell migration and cytoskeletal rearrangements. Activation of Rac1 has emerged as an important event in the stimulation of cytoskeletal rearrangements, migration and invasion by uPAR engaged with Vn (Kjoller and Hall 2001). Smith et al. provided insights into the downstream uPAR signalling leading to Rac1 activation through the DOCK180 guanine nucleotide exchange factor in the BE colon carcinoma cell line. The functional cooperation with *β*_3_ integrin leads to the formation of the p130Cas–CrkII signalling complex, resulting in a mesenchymal-type morphology, cell migration and invasion (Smith et al., 2008). Following uPA binding, the uPAR stimulates MCF-7 cell migration that is inhibited by the MEK-specific antagonist PD098059, as well as the Y-27632 antagonist of the Rho-Rho kinase pathway, suggesting a cooperation between these two pathways to promote cell migration (Jo et al., 2002). Sturge et al. reported that uPAR-directed chemotaxis of human breast cancer MDA-MB231 cells involves the activation of Cdc42, Rac1 and translocation of N-WASP to the actin cytoskeleton (Sturge et al., 2002). In vascular muscle cells, RhoA and Rac1, but not Cdc42, are directly associated with Tyk2 and PI3-K and mediate uPA/uPAR-dependent signalling leading to cell migration (Kiyan et al., 2003). Margheri et al. reported that uPAR association with β2 integrin drives the activation of Rac1 and Cdc42 activation in microvascular endothelial cells (Margheri et al., 2006). In podocytes, the uPAR is required to activate αvß3 integrin, in turn, promoting activation of Cdc42 and Rac1 and leading to increased migration (Wei et al., 2008). Vial et al. showed that colon carcinoma cell migration is regulated by Fra-1 transcription factor that inactivates ß1 integrin subunit and down-regulates RhoA activity. The reduction of RhoA activity favors uPAR signalling leading to Rac activation, lamellipodia extension and migration (Vial et al., 2003). In prostate and melanoma cells, the uPAR controls the mesenchymal-type movement as well as the amoeboid-type, characterised by a RhoA-directed rounding of the cell body, formation of a cortical ring of actin and reduction of Rac-1 activity. Upon uPAR silencing or following cell exposure to a peptide inhibiting uPAR-β1/β3 integrin association, both types of motion are markedly reduced (Margheri et al., 2014). The relationship between uPAR and Rho family GTPases seems to involve reciprocal controls: constitutively active RhoA stimulates uPAR transcription in NIH-3T3 cells, while Rac1 does not (Muller et al., 2000). In murine fibroblasts, the extent of active Rac1 depends on the uPAR expression level, as shown by studies with in uPAR+/+ and uPAR−/− embryonic fibroblasts. In the same study, the authors show that LRP-1 is an important regulator of Rac1 activation, in a uPAR-dependent manner (Ma et al., 2002). We have uncovered a previously unknown role for RhoB as a key mediator of uPAR-dependent responses in prostate cancer cells, including cell migration, invasion and adhesion to Vn. The full uPA causes RhoB activation and increases its expression, in a proteolytic-independent manner (Alfano et al., 2012; Ridley 2013).

The uPAR is indeed a regulator of focal adhesion contact stability, as silencing of uPAR causes a disassembly of the focal adhesion molecules, such as FAK, Paxillin and p130Cas and reduces phospho-FAK levels in medulloblastoma cells (Nalla et al., 2010). Studies on oral squamous carcinoma cells show that uPAR overexpression is associated to focal adhesion proteins expression and phosphorylation, suggesting a model in which the engagement of uPAR/α3β1/laminin-5 leads to phosphorylation of p130cas and Cdc42 by c-Src tyrosine kinase and modulation of focal adhesion dynamics (Shi et al., 2011).

Several reports show that the uPAR is relevant in the control of cell survival. We have shown that the stable reduction of uPA or uPAR expression by RNA interference leads to an increased susceptibility to UV-, cisplatin-, and detachment-induced apoptosis (anoikis). These effects are mediated by Bcl-xL transcriptional activation through the MEK/ERK- and phosphatidylinositol 3-kinase/Akt-dependent pathways (Alfano et al., 2006). The possibility that the uPA/uPAR interaction may counteract or promote pro-apoptotic signals is shown by Dumler’s group in human mesangial cells, through the activation of ERK, Akt and BAD signalling. (Tkachuk et al., 2008). In another study, the downregulation of uPA and uPAR by RNA interference in two MDA-MB 231 and ZR 75-1 breast cancer cell lines results in the overexpression of pro-apoptotic caspases (Subramanian et al., 2006). Others report that uPAR signalling through PDGFR-β controls the Bcl-2/Bax ratio, thus regulating mitochondrial mediated apoptosis (Malla et al., 2010). In glioblastoma, ligand-engaged uPAR leads to the repression of the Bim proapoptotic factor (Wykosky et al., 2015).

## Epithelial to Mesenchymal Transition

**FIGURE 2:**
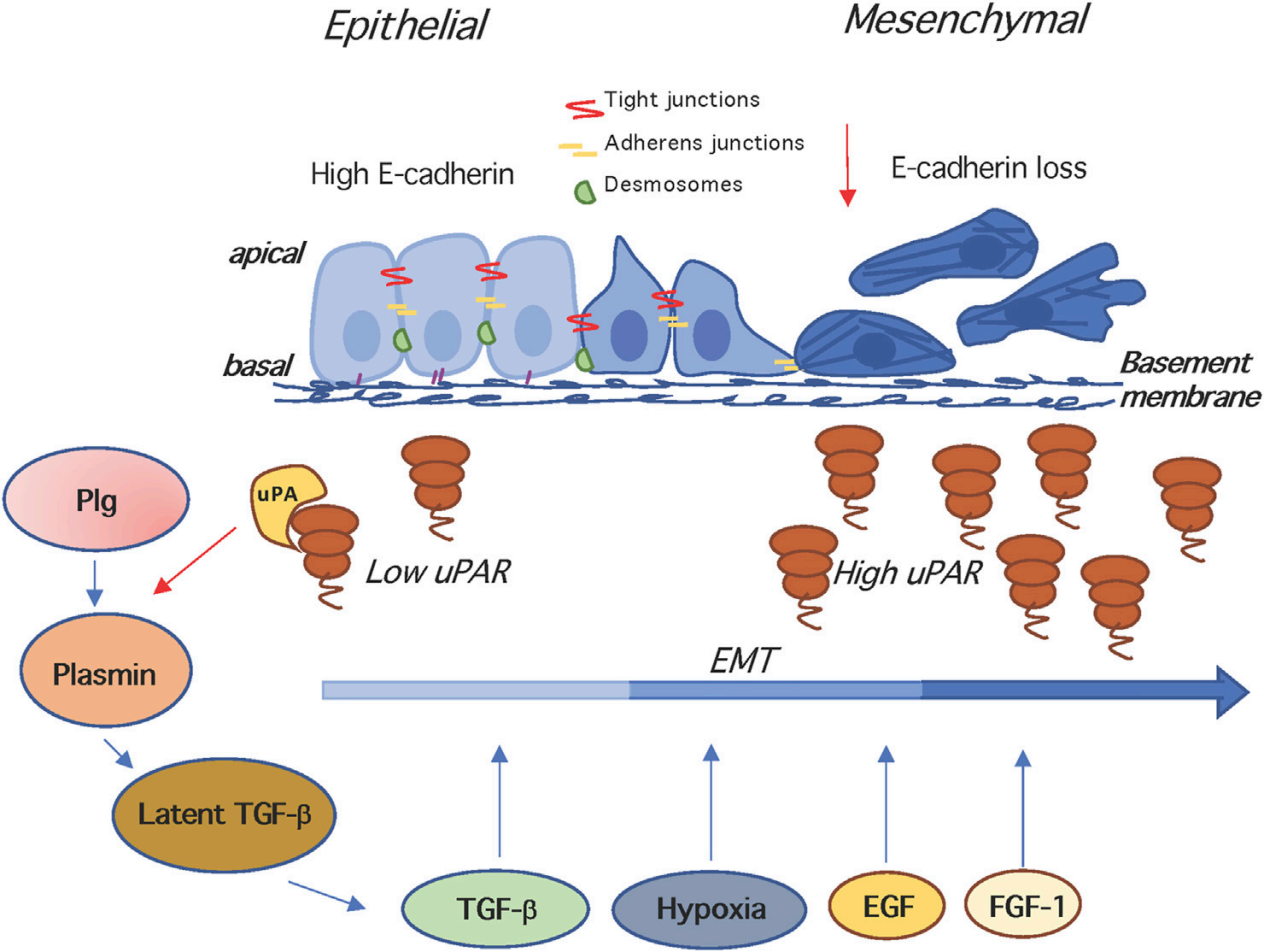
The uPAR and epithelial to mesenchymal transition. In the EMT, epithelial cells lose their junctions and apical–basal polarity, reorganize their cytoskeleton, change their motility/adhesion properties and reprogramme gene expression. EMT is induced by hypoxia, and growth factors like TGF-β, EGF, FGF-1, disrupting of cell-cell junctions and inducing loss of E-cadherin. The uPA- and Plg-dependent activation of latent TGF-β generates active TGF-β that is a potent EMT inducer. Both TGF-β and uPA/uPAR are involved in the induction of EMT, and mutual cooperation may be operating, since TGF-β stimulates the expression of uPA and uPAR, and the enhancement of uPA levels increases plasminogen activation, which in turn activates matrix-associated latent complexes. Plg, plasminogen; TGF -β, Transforming growth factor beta.

During epithelial to mesenchymal transition (EMT), epithelial cells lose their junctions and apical–basal polarity, reorganize their cytoskeleton, undergo changes in the signalling programmes that define cell shape and reprogramme gene expression; this increases also the motility of individual cells and enables the development of an invasive phenotype (Nieto et al., 2016) (Figure 2). EMT is essential for numerous developmental processes including mesoderm ad neural tube formation. It is noteworthy that tumour cell invasion shares many phenotypic similarities to EMT, therefore the capacity of cancer cells to undergo EMT is now considered a hallmark of tumor progression (Dongre and Weinberg 2019). Many oncogenic signalling mediators, like Src, Ras, Ets, integrins, Wnt/β-catenin and Notch are known to induce EMT. Among these, Snail and Slug, transcriptional repressors of E-cadherin, are activated by the Ras-MAPK-dependent pathway (Kalluri and Weinberg 2009). In early studies, causally linking the uPAR to EMT, Zhang and coworkers demonstrated that uPAR overexpression leads to a mesenchymal transition of kidney epithelial cells expressing α3ß1 integrins (Zhang et al., 2003). The first evidence that uPAR-dependent signalling is involved in hypoxia-induced EMT of breast cancer cells was published by Lester et al. (Lester et al., 2007). Gonias’s group confirmed that the phenotypic and signalling changes associated to EMT in MDA-MB-468 breast cancer cells are consequent of hypoxia-induced uPAR expression and signalling. In fact, hypoxia-induced disruption of cell-cell junctions and loss of E-cadherin from cell surface is blocked by uPAR silencing and mimicked by uPAR overexpression in normoxia. These findings imply that uPAR-initiated cell signalling may be targeted to counteract EMT in cancer (Jo et al., 2009). Evidence that uPA or uPAR targeting reduces hypoxia-induced cell EMT, invasion and migration was obtained in medulloblastoma tumors (Gupta et al., 2011).

The overexpression of uPAR has been linked to EMT in several reports (Figure 2): Huang et al. show that the enhancement of uPAR transcription by the Forkhead box M1 (FOXM1) factor contributes to pancreatic tumor EMT and metastasis (Huang et al., 2014). Furthermore, in nasopharyngeal carcinoma, uPAR overexpression promotes not only migration and invasion but also EMT (Bao et al., 2014). The relevance of uPAR to the EMT process in SGC-7901 and BGC-823 human gastric cancer cell lines is shown by the finding that uPAR silencing significantly reduced EMT induction by EGF (Wang et al., 2017). Moreover, silencing the uPAR results in the inhibition of melanoma EMT stimulated by the conditioned media of mesenchymal stem cells or by TGFß (Laurenzana et al., 2015). Hepatocellular carcinoma cell lines expressing high levels of CD133+, exhibit a concomitant upregulation of invasion-associated genes like uPAR, MMP1, MMP2, and EMT regulators like Snail and Twist, suggesting that the expression of CD133+ is associated to a subtype of aggressive hepatocellular carcinoma (Na et al., 2011).

Increased uPAR expression is involved in the promotion of EMT in chronic obstructive pulmonary disease (Wang et al., 2013). In contrast, another report rules out the involvement of uPAR in benzylisothiocyanate-mediated inhibition of EMT, at least in MDA-MB-231 and SUM159 breast cancer cells (Sehrawat et al., 2013).

In neuroblastoma cells, uPAR expression is essential for maintaining the epithelial phenotype and genetic deletion of uPAR by CRISPR/Cas9 technology promotes EMT, increasing cell migration and proliferation (Rysenkova et al., 2018; Semina et al., 2020). In a FGF-induced EMT model, the uPAR is immediately up-regulated and maintained throughout FGF-1 stimulation; in this context, by genome-wide oligoarray technology, uPAR is identified as immediate FGF/FGFR-dependent EMT biomarker, which might be a prognostic factor for bladder carcinoma tumor progression (Billottet et al., 2008). Conversely, the ectopic expression of the ERp29, a molecular chaperone that plays a critical role in protein secretion, results in G0/G1 arrest of MDA-MB-231 cells, causes EMT and suppresses tumor growth in nude mice, also by inhibiting uPAR transcription (Bambang et al., 2009).

## Cell Fate Specification and Differentiation

A fundamental characteristic of stem cells is their lasting ability to multiply and differentiate into specialized cells that can no longer divide. Cancer stem cells (CSCs), also known as tumor-initiating cells (TICs) contribute to recurrence, heterogeneity, metastases, multidrug resistance and radiation resistance, due in part to their ability to self-renew and differentiate into heterogeneous lineages of cancer cells. One early evidence suggesting a link between the uPAR and the stem cell-like phenotype in tumors emerged from the gene expression profiling of rat fetal hepatoblasts, adult hepatocytes and human hepatocellular carcinomas (HCC). The resulting signatures show the upregulation of invasion and metastasis-related genes, such as the uPAR, VIL2 (encoding ezrin), and CD44 in hepatoblasts and in a subtype of HCC associated to poor prognosis (Lee et al., 2006). Subsequently, Gutova et al. reported that in six small cell lung cancer cell lines, the clonal expression of uPAR is associated to multidrug resistance, high clonogenic activity and co-expression of CD44 and MDR1, putative cancer stem cell markers. This suggests that uPAR + cells may define a specific subpopulation of cells to be targeted in small cell lung cancer (Gutova et al., 2007). Accordingly, a recent study showed that uPAR deletion by the CRISPR/Cas9 technique reduces the multidrug resistance of colon and adenocarcinoma tumor cells (Wang et al., 2019).

The ability of uPAR to induce CSC–like properties has largely emerged. Following ionizing radiation treatment, uPAR overexpression leads to increased WNT-7a-β-catenin-TCF/LEF-mediated transactivation in UW228 and D283 medulloblastoma cell lines, thereby promoting cancer stemness (Asuthkar et al., 2012). In glioma cell cultures, silencing of uPAR and cathepsin B downregulates the expression of CD133, Nestin, Sox2 and Bmi1 and reduces the number of glioma-initiating cells (Gopinath et al., 2013). Keasey et al. found that blood-derived Vn rapidly and potently activates interleukin 6 (IL-6) and leukemia inhibitory factor (LIF), known to promote self-renewal, *in vitro* and after vascular injury in the brain, unless the uPAR is pharmacologically inhibited (Keasey et al., 2018). In contrast, recent studies by Laurenzana’s group in melanoma and colon cancer cell lines showed that knocking out uPAR gene by the CRISPR/Cas9 technique results in growth inhibition, with the concomitant appearance of stemness markers (Biagioni^a^ et al., 2021). In human mammary MDA-MB-468 cells, uPAR overexpression induces a CD24−/CD44 + phenotype, characteristic of CSCs, and upregulates cell surface expression of integrin subunits β1/CD29 and α6/CD49f, putative stem cell biomarkers. It has to be remarked that in a mouse orthotopic breast cancer model, the uPAR overexpression enhances mammosphere formation in culture and tumor development (Jo et al., 2010) (Figure 2). The combined silencing of uPAR and cathepsin B in CD133 + TICs leads to the dissociation of pPKC θ/δ, integrin β1 and PKC ζ, integrin β1 complex as well as the dissociation of FAK, vinculin and α-actinin, thus inhibiting PKC/integrin signalling, and ultimately controlling GBM tumor invasion and resistance (Alapati et al., 2012). Moreover, uPAR-expressing GBM cells show a mesenchymal-type gene signature, an increased capacity for cell survival, together with stem cell-like properties (Gilder et al., 2018). Another report highlights the relationship between uPAR expression and ability to form spheres as well as transplantable tumors. In particular, spheres derived from the H446 SCLC cell line exhibit an increased proportion of uPAR and CD133 expressing cells, associated to *in vivo* clonogenic, tumorigenic and drug-resistant properties. The H446 SCLC uPAR + cells can be differentiated to CD56^+^, CK+, uPAR-, supporting the existence of a tumor sphere-forming stem cell population (Qiu et al., 2012). In a study focused on the chemoresistance of malignant pleural mesothelioma (MPM), the uPAR, together with CSC markers Bmi-1, ABCG2 and CD133, exhibit a hypoxia-dependent expression, a hallmark for the selection of chemoresistant cells. The expression of uPAR, together with Bmi-1, ABCG2 and CD133, confers chemoresistance to cisplatin and pemetrexed to the MPM cell lines, identifying a population of putative drug-resistant CSC to be, possibly, targeted in anti-cancer therapies (Cortes-Dericks et al., 2010).

It is known that the formation of *ß*-catenin–LEF-1 complexes can promote EMT, associated to the loss of cell–cell adhesion and acquisition of the mesenchymal phenotype. Asuthkar et al. showed that in the UW228 and D283 medulloblastoma cell lines, uPAR overexpression leads to increased WNT-7a-β-catenin-TCF/LEF-mediated transactivation whereas uPAR silencing has an opposite effect, uncovering a mutual regulatory relationship between uPAR and WNT/β-catenin signalling (Asuthkar et al., 2012). The connection between migration of bone marrow precursor cells and the uPAR is shown in many studies. In particular, the SuPAR or the uPAR_84-95_ chemotactic peptide stimulated migration of human CD34 ^+^ HSCs and inactivated CXCR4, the chemokine receptor primarily responsible for HSC retention in bone marrow (Selleri et al., 2005). In mice, i. p. administration of the uPAR_84-95_ chemotactic sequence induces an increase of CD34 ^+^ HSCs/HPCs in peripheral blood, comparable to that of G-CSF, suggesting potential clinical applications in HSC transplantation (Selleri et al., 2006).

An important property of hematopoietic stem and progenitor cells is the modulation of cell adhesion in the osteoblastic niche, allowing their differentiation to the proliferating cell stage. In the murine system, the uPAR has been shown to be expressed by a subset of hematopoietic/stem progenitor cells and be essential for their homing engraftment and mobilisation (Tjwa et al., 2009). In the hypoxia-enhanced adhesion of HSPCs (hematopoietic stem progenitor cells) to the bone marrow niche, it is reported that an uPAR-mediated pathway, involving the soluble form of LR11 lipoprotein membrane protein, stabilizes the hematological pool size by controlling cell adhesion to the bone marrow niche (Nishii et al., 2013). The expression of uPAR is reported to be undetectable in CD33 ^+^ myeloid precursors, CD14 ^+^ monocytic cells. In humans, G-CSF treatment induces the upregulation of uPAR on circulating CD33^+^ and CD14 ^+^ cells, and uPAR shedding leading to the appearance of serum SuPAR (Selleri et al., 2005). Early research on uPAR protein levels in myelomonocytic cells differentiating to a macrophage-like phenotype, showed that the uPAR undergoes a 50-100 fold upregulation in the U937 cells exposed to phorbol esters or to TGFß/Vitamin D3 (Stoppelli et al., 1985). Later, it was shown that inhibition of the uPA/uPAR interaction prevents adhesion and cysteine proteinase activity, both markers of myeloid differentiation (Nusrat and Chapman 1991). These data are in agreement with those by Sloand, showing that the uPAR plays a fundamental role as a differentiation antigen on cells of the myelomonocytic lineage and as an activation factor for monocytes and T lymphocytes (Sloand 2005). More recent data indicate that uPA stimulates the differentiation of monocytes into macrophages, resulting in prolonged macrophage survival in atheroschlerotic lesions and accelerating lesion development (Paland et al., 2013).

Up- or down modulation of uPAR expression is causally involved in the differentiation of smooth muscle cells into vascular smooth muscle cells, as monitored by changes in cell morphology and expression of specific marker proteins (Vallabhaneni et al., 2011). In the osteogenic differentiation process from mesenchymal stem cells, the uPAR is a mediator of differentiation and propagation of the osteogenic process via interference with the complement C5a receptor; uPAR also determines the progression of vascular calcification in chronic cardiovascular inflammation *in vivo* (Kalbasi Anaraki et al., 2014). Anaraki et al. recently reported that osteclastogenesis is impaired in co-cultures of monocyte-derived osteoclasts and in osteoblasts derived from uPAR deficient mesenchymal stem cells, showing that uPAR directly mediates osteoclast formation and differentiation via PI3K/Akt/NF-kB pathway (Kalbasi Anaraki et al., 2015).

## Bacterial and Viral Infections

The relationship between the uPAR and viral pathogenetic mechanisms has been the subject of many studies. In the early 1990s, it was reported that HIV-1 infection causes the upregulation of uPAR cell surface expression in monocytes and T lymphocytes *in vitro* and *in vivo* (Nykjaer et al., 1994). Whether HIV infection enhances uPAR expression levels by a direct mechanism or indirectly through the effect of proinflammatory cytokines remains to be determined. As reported by Alfano et al., uPAR engagement with uPA inhibits HIV-1 expression in U937-derived chronically infected promonocytic U1 cells, suggesting a functional similarity of uPA signalling with antiviral agents mimicking IFNs in their inhibition of HIV expression and replication (Alfano et al., 2002).

The protective role of uPAR against bacterial infections is further shown by Rijneveld et al., demonstrating that uPAR is crucial for adequate recruitment of neutrophils in the mice lungs during infection by S. pneumoniae, via a mechanism dependent on *β*_2_ integrin (Rijneveld et al., 2002). The relevance of uPAR to the lymphocyte recruitment to the lungs is shown by the impaired migration of uPAR−/− lymphoblasts in pulmonary infections (Gyetko et al., 2001). The efficient induction of uPAR expression in T lymphocytes is reported to occur following co-clustering of β1 or β2 integrins with the antigen receptor complex, and involving both PKC activation and increased intracellular cyclic AMP (Bianchi et al., 1996). In neutrophils, Factor XII regulates cell adhesion, migration, and release of neutrophil extracellular traps in a process called NETosis and its deficiency is associated with decreased migration. Recent studies have shown that Factor XII signals through uPAR-mediated Akt2 phosphorylation at S^474^, with the involvement of αMβ2 integrin (Stavrou et al., 2018; Renné and Stavrou 2019). Many reports address the relevance of plasma levels of SuPAR in infectious diseases. In HIV infection, the enhanced cell surface expression of uPAR on monocytes and T-lymphocytes *in vitro* and *in vivo*, may lead to an increased shedding into serum. In one study, the authors showed that uPAR overexpression in patients with advanced HIV-1 disease, is associated to high serum levels of SuPAR and poor overall survival and mortality rates (Sidenius et al., 2000). In severe HIV infections, the SuPAR plasma levels are indicators of the metabolic syndrome, a condition in which a group of risk factors for cardiovascular disease and type 2 diabetes occur together (Andersen et al., 2008). Furthermore, it was shown that engaged uPARs trigger the inefficient release of HIV from infected monocytic cells, and that this mechanism could be inhibited by interfering with uPA/uPAR interaction, Mac-1 integrin activation, or prevention of its association with uPAR (Alfano et al., 2009). Others provide new mechanistic insights into how engaged uPARs may enhance HIV virions accumulation in intracytoplasmic vesicles by RhoA- and PKCε-dependent pathways in promonocytic U1 cells (Graziano et al., 2011). Moreover, in chronically infected promonocytic U1 cell line, Nebuloni and coworkers demonstrated that the cleaved SuPAR form from HIV-infected tonsil histocultures is endowed with the ability to inhibit migration and induce virus expression (Nebuloni et al., 2013).

High SuPAR levels are found not only in the plasma/serum of HIV-infected individuals, but also in the central spinal fluid of patients with neurological complications (Sidenius et al., 2004; Sporer et al., 2005; Nebuloni et al., 2009). In contrast, only minor differences were observed between wild type and uPAR null mice, infected with the HRSV influenza virus, indicating that the uPAR does not play a major role neither in the modulation of virus replication nor in the innate immune response against influenza infections *in vivo* (Ramos et al., 2015). The process of virion assembly, budding, and release from the plasma membrane has been very well characterized; it is known that in T lymphocytes the HIV virions budding occurs in lipid rafts whereby host cell cholesterol, sphingolipids, and GPI-linked proteins are incorporated into the viral envelope (Nguyen and Hildreth 2000; Ono and Freed 2001). Increasing research has focused on the interplays between Ly6/uPAR family of GPI-anchored proteins and viral pathogens, and the results have provided new insights into viral entry and virus-host interactions (Yu et al., 2019). In Mar et al. the authors showed that LY6E, a key member of the LY6/uPAR family, belongs to a class of IFN-inducible host factors that enhance viral infectivity without suppressing IFN antiviral activity (Mar et al., 2018).

Given the well-known involvement of uPAR in fibrinolysis, inflammation, and immunity, many scientists focused their recent research on its potential role in coronaviruses infection and related consequences, with particular regard to SARS-CoV-2. First of all, the increased transmissibility of Covid-19 is caused also by an inserted furin site in SARS-CoV-2 spike (S) protein, that is cleaved during virus entry. Recent data show that plasmin is able to cleave the S protein furin site, thus favoring SARS-CoV-2 infection (Hou et al., 2021). Second, the Covid-19 patients often suffer from a prothrombotic state associated to severe coagulopathies, that is not fully investigated. In the lungs of Covid-19 patients, the mRNA levels for regulators of the uPA and uPAR-dependent pathways are altered, suggesting that this may lead to abnormal fibrin deposition (Mast et al., 2021). Furthermore, Covid-19 patients show dramatically elevated SuPAR blood levels that may be directly involved in the Covid-19–related acute kidney injury (AKI) (Rovina et al., 2020). Azam and coworkers found that blood SuPAR levels in patients hospitalized for Covid-19 are predictive of incident AKI (Azam et al., 2020). This is not surprising, in view of the finding that high SuPAR levels in serum may cause Focal Segmental Glomerulosclerosis (FSGS), a severe proteinuric kidney disease (Wei et al., 2011). Evidence suggesting a pathological role for the uPAR isoform 2, that includes D1 and half of D2, is obtained in mice exhibiting signs of severe renal disease similar to FSGS (Wei et al., 2019).

Blood biomarkers capable of risk stratification are of great importance in effective triage and critical patients care. It has been reported that uPAR represents a biomarker of disease progression, and its levels well correlate with comorbidities associated with the death of coronavirus patients (Chalkias et al., 2020; de Bruin et al., 2021). In severe Covid-19 patients with acute respiratory distress syndrome (ARDS), a recent study reports high plasma levels of SuPAR, that is expressed by an expanded population of myeloid cells. In contrast, low SuPAR levels are associated to a specific immune transcriptome and to favorable clinical outcomes. In a recent report, the SuPAR is identified as a marker indicating a state of hyperinflammation and hypercoagulation for patients risk stratification (Sarif et al., 2021). In another study, elevated SuPAR levels identified patients needing an early targeted treatment with anakinra, a recombinant IL-1 receptor antagonist. Following early identification and treatment of these patients, a 70% decrease in the relative risk of disease progression to respiratory failure and a reduction in mortality with anakinra treatment was observed (Kyriazopoulou et al., 2021). In conclusion, the uPAR activity may directly or indirectly influence SARS-Cov-2 pathogenetic mechanism and its consequences by several means, including S protein cleavage, fibrinolytic balance, and blood SuPAR levels (D’Alonzo et al., 2020). As a circulating marker, the SuPAR can be instrumental to stratify patients at risk to undergo severe illness and define early their therapeutic needs.

## Diagnostic and Terapeutic Aspects

The pleiotropic function of uPAR and its involvement in many distinct human diseases has encouraged many investigators to apply the detailed molecular knowledge generated in past three or more decades to the clinical practice. Consolidated evidence shows that, in aggressive malignancies, the uPAR is often overexpressed and associated to high risk of relapse and unfavourable clinical outcome (Li Santi et al., 2021). Currently, uPAR expression and distribution are regarded as a tool to be developed for prognostic and diagnostic purposes, as well as an attractive therapeutic target in the management of neoplastic conditions.

The overexpression of uPAR in the invasive tumor regions and their relative adjacent microenvironment has encouraged its targeting with high affinity and specificity compounds in non-invasive diagnostic imaging. One such example is the uPAR-binding AE105 peptide, that was traced with ^64^Cu and successfully tested in mice by microPET for its specificity to target uPAR-bearing U87 glioblastoma cells (Li et al., 2008). Recently, a phase 1 clinical trial using AE105 has been completed in patients with breast, prostate, and bladder cancers, establishing that administration of this agent is safe and results in a favorable biodistribution and stability (Persson et al., 2015; Skovgaard et al., 2017). Further studies were devoted also to develop new uPAR-targeted optical probes for fluorescence-guided surgery, initially characterized in nude mice with patient-derived glioblastoma xenografts, as candidates for translation into human use (Kurbegovic et al., 2021). Among other uPAR-related agents for *in vivo* tumor imaging is the Cy5.5-labeled monoclonal antibody, specifically detecting free and occupied uPAR in orthotopic mammary carcinomas in mice (Dullin et al., 2009)*.* Li et al., authored a comprehensive review on the uPAR as a target for *in vivo* imaging and therapy (Li et al., 2013).

As described earlier in this review, the uPAR is detected not only in tissues, but is found in its soluble form or SuPAR, in body fluids, like urine, plasma, blood, serum, and cerebrospinal fluid. Because SuPAR expression has been reported to correlate with disease severity in cancer, arthritis, liver fibrosis, malaria, and bacterial infection, its concentration in blood is being taken into consideration to assist clinical decision-making. In diagnostic studies for non-invasive early recognition of cancer, the level of SuPAR in the blood circulation is significantly associated with cancer diagnosis in patients with non specific symptoms of cancer, compared to disease-free patients (Rasmussen et al., 2017). Another recent study on prognostic markers for metastatic colorectal cancer shows the association of SuPAR serum levels, as determined with enzyme-linked immunosorbent assay, with overall patients survival (Blomberg et al., 2021). Furthermore, the SuPAR may predict response to therapy in colorectal cancer patients, as patients with low levels of circulating suPAR and a wild-type KRAS tumor benefit from treatment with oxaliplatin and cetuximab, as compared to patients with wild-type KRAS and high levels of SuPAR (Tarpgaard et al., 2015). Examples from non-neoplastic pathological conditions associated to enhanced SuPAR expression include the focal segmental glomerulosclerosis, a disease in which high SuPAR plasma levels predict disease progression to end-stage renal failure (Winnicki et al., 2019). It was recently reported that high levels of blood SuPAR predict severe/critical Covid-19 disease and are associated with length of hospital stay (Enocson et al., 2021).

Evidence accumulated in the past 3 decades suggests that uPAR is an attractive target for therapeutic intervention to counteract cancer invasion and metastases. Based on the information gathered on uPAR structure, also complexed with vitronectin and uPA, many distinct molecules interfering with uPAR interactions and, ultimately, function have been designed and tested throughout the years. First of all, seminal studies established that most of the binding ability of uPA is retained by a peptide spanning residues 12–32 (GFDp) of the human sequence (Stoppelli et al., 1985; Appella et al., 1987). Because the important residues, Lys23, Tyr24, Phe25, IIe28, and Trp30 are located within the same domain, inhibition of uPAR binding by a single peptide is a feasible strategy (Magdolen et al., 1996). Synthetic cyclic peptides covering the residues 19–31 were reported to be potent inhibitors of uPA/uPAR association, surface-associated plasminogen activation and fibrin degradation (Magdolen et al., 2001). Other uPA-derived cyclic peptides like WX-360 derived from the previous ones inhibit the spreading of ovarian cancer cells in the mouse (Sato et al., 2002).

For uPAR targeted-toxin therapy, the receptor binding region of uPA (ATF, residues 1–135) was fused with saporin and the chimeric fusion protein displayed a specific toxic effect in uPAR-expressing bladder cancer xenografted cells, suggesting promising cytotoxic treatements (Fabbrini et al., 1997; Zuppone et al., 2020).

Another approach was to conjugate a uPAR-specific targeting peptide onto magnetic nanoparticles for the development of theranostic agents for diagnosis and image-guided therapy of uPAR-overexpressing primary and metastatic tumor lesions (Hansen et al., 2013).

The multiple studies on the uPAR chemotactic sequence led to the generation of many distinct uPAR_88-92_ -derived peptides inhibiting uPAR signalling. In the stimulatory SRSRY peptide, Ser substitutions, like in pERERY-NH2, generate antagonists blocking uPAR-dependent cell migration and signaling by preventing with uPAR/FPR interaction (Bifulco et al., 2008). Other peptides, like the Ac-Arg-Glu-Arg- Phe-NH2 peptide (denoted as RERF) reduce lung metastasis number and size in nude mice (Carriero et al., 2009). Further FPR antagonists include the RI-3 peptide and suggest a pharmacophore inhibitor model for further development of anti-invasive agents (Minopoli et al., 2019). Among antibodies proposed for direct inhibition of uPAR engagement with uPA, the humanized ATN-658 MoAb, inhibiting both metastasis and tumor proliferation in mouse models, also in combination with zolendronic acid, has emerged as a new promising tool for clinical trials (Mahmood et al., 2020).

Previous work from our laboratory indicated that the main uPAR ligand, uPA associates to av integrin through its connecting peptide region (CP, residues 132–158), thus bridging uPAR and the αvβ5 (Franco et al., 2006). Peptides derived from this region are endowed with the ability to modulate cell migration: in particular, the 135–143 peptide is a strong inhibitor at picomolar concentrations (Franco et al., 2013). A thourough conformational analysis of the CP-derived, anti-migratory peptides suggested the design of a novel cyclic peptide denoted uPAcyclin, corresponding to the N-terminal region of CPp, with the S138E substitution in a stabilized, putative bioactive conformation. The uPAcyclin has anti-migratory and anti-invasive properties in culture and prevents lung metastases in nude mice through its interaction with the αv-integrin subunit (Belli et al., 2020). Furthermore, the novel peptide induces a partial reversion of the Cancer-Associated Fibroblasts (CAF) phenotype and markedly reduces the pro-invasive ability of peritumoral CAFs from breast cancer patients in combination with MDA-MB-231 mammary adenocarcinoma cells in organotypic assays (De Vincenzo et al., 2019; Belli et al., 2020). Independent evidence showed the multiple activities of an 8-mer linear peptide corresponding to residues 136–143 of human uPA and denoted Å6. This peptide, sharing most of the sequence with uPAcyclin, exhibits anticancer and anti-metastatic effects in tumor cell cultures and mouse models in many preclinical studies (Guo et al., 2000). Among the activities displayed by Å6, are a remarkable cytotoxic activity for chronic lymphocytic leukemia cells and inhibition of VE-cadherin degradation and alterations of the blood retinal barrier in diabetic rats (Navaratna et al., 2008). Several clinical studies have shown that Å6 is well tolerated, with no toxicity (Berkenblit et al., 2005). It is noteworthy that Å6 reached phase II clinical trials for treatment of ovarian epithelial, fallopian tube, or primary peritoneal carcinomas (Ghamande et al., 2008; Gold et al., 2012).

Another approach that is worth to be mentioned is the use of oncolytic viruses, that selectively infect and replicate in tumor cells, inducing antitumor immunity. Jing et al., published that oncolytic measles virus targeting stromal uPAR and CD46 in colon cancer cells results in enhanced antitumor effects, supporting preclinical and clinical development of therapies based on stroma-directed systemically administered oncolytic viruses (Jing et al., 2020).

However, despite the advances in the molecular design and generation of uPAR-derived peptides or novel interactors, no uPAR-targeting therapeutics have currently progressed to clinical trials (Metrangolo et al., 2021). Most studies are at preclinical stages and further analyses of the biodistribution, toxicity profile, pharmacokinetics of the novel agents *in vivo* are needed to assess their potential benefits in a clinical setting.

## Concluding Remarks

The complex interactions between uPAR ligands and co-receptors result in profound changes of cellular phenotype, including the modulating of cell migration, survival, adhesion, invasion. Cell signaling pathways activated downstream of uPAR have been shown to be crucial in a variety of physiological and pathological processes *in vivo*, including many human diseases. Beyond the well consolidated proteolytic-dependent and independent activites of uPAR in tumor invasion and metastases, this review covers the current knowledge on the role of this receptor in the epithelial to mesenchymal transition, in cell fate and differentiation as well as in infectious diseases, including Covid-19. Further information about the involvement of the uPA/uPAR system in human pathologies may come out in the future, and we expect that the profound knowledge of the molecules, interactors and signalling involved will support uPAR-related diagnostic and therapeutic applications in human diseases.

## Abbreviations

ECM, extracellular matrix; EMT, epithelial to mesenchymal transition; uPA, urokinase-type plasminogen activator; uPAR, urokinase receptor
